# Factors Influencing Parental Decision-Making in Pediatric Cochlear Implantation: A Narrative Review

**DOI:** 10.7759/cureus.102714

**Published:** 2026-01-31

**Authors:** Tyler A Rosenbluth, Harvey N Mayrovitz

**Affiliations:** 1 Medicine, Nova Southeastern University Dr. Kiran C. Patel College of Osteopathic Medicine, Fort Lauderdale, USA; 2 Medicine, Nova Southeastern University Dr. Kiran C. Patel College of Allopathic Medicine, Davie, USA

**Keywords:** caregiver perspectives, cochlear implantation, deaf identity, family-centered counseling, health literacy, parental decision-making, pediatric hearing loss, socioeconomic barriers

## Abstract

Parental decision-making regarding pediatric cochlear implantation (CI) is shaped by a complex set of emotional, cultural, informational, and socioeconomic factors that extend well beyond clinical candidacy and expected auditory outcomes. This narrative review synthesizes findings from 25 peer-reviewed studies published between 2000 and 2025 to describe how parents and caregivers navigate this high-stakes process and weigh potential benefits against uncertainties related to long-term outcomes, rehabilitation needs, and their child’s future communication identity. Across studies, families reported challenges in understanding complex medical information, managing the emotional burden of early, time-sensitive decisions, and accessing consistent support from healthcare professionals. Structural influences, including financial strain and variability in service availability, further contributed to delays or hesitations in decision-making. Despite these obstacles, many parents expressed a desire for clear guidance, culturally sensitive counseling, and accessible educational resources to help them make decisions aligned with their values and expectations. By highlighting recurring themes across diverse settings, this review underscores the importance of a more holistic, family-centered approach to counseling and support in pediatric CI.

## Introduction and background

Cochlear implantation (CI) is a transformative intervention for children with severe to profound sensorineural hearing loss, offering access to spoken language and auditory development during critical periods of neuroplasticity [1]. While its clinical effectiveness is well documented, particularly when implantation occurs early in life, the decision to pursue this intervention is complex and multifaceted [2]. For parents and caregivers, the process often involves navigating not only medical recommendations but also emotional, cultural, and practical concerns while making high-stakes decisions on behalf of their child.

Factors beyond audiological thresholds or candidacy criteria shape this decision-making process. Caregivers may contend with feelings of uncertainty, fear of long-term consequences, and limited time to act within developmental windows. These challenges are often exacerbated by conflicting input from medical providers, educators, and Deaf community members, as well as evolving family beliefs about identity, language, and communication modality [3]. For some families, the lack of clear, consistent guidance adds to the emotional burden of navigating an already difficult choice.

Although much of the CI literature has focused on clinical outcomes, device performance, and speech and language gains, an emerging body of work emphasizes the role of sociocultural context, emotional readiness, and systemic barriers in shaping decision-making [4]. These influences are particularly pronounced among families facing socioeconomic challenges, including limited insurance coverage, geographic isolation, or inadequate access to follow-up services. Such disparities can delay care, diminish confidence in the process, or lead to decisional regret.

Despite increasing awareness of these issues, no review to date has comprehensively synthesized the emotional and socioeconomic factors that influence parental decision-making regarding pediatric CI. This narrative review aims to synthesize existing literature on the emotional, cultural, informational, and socioeconomic factors that influence parental decision-making regarding pediatric CI and map the lived experiences of parents and caregivers. Through identifying recurring challenges, motivators, and unmet needs, this work seeks to inform more compassionate and equitable approaches to family counseling, education, and long-term support. 

## Review

Study design and reporting framework

**Figure 1:**
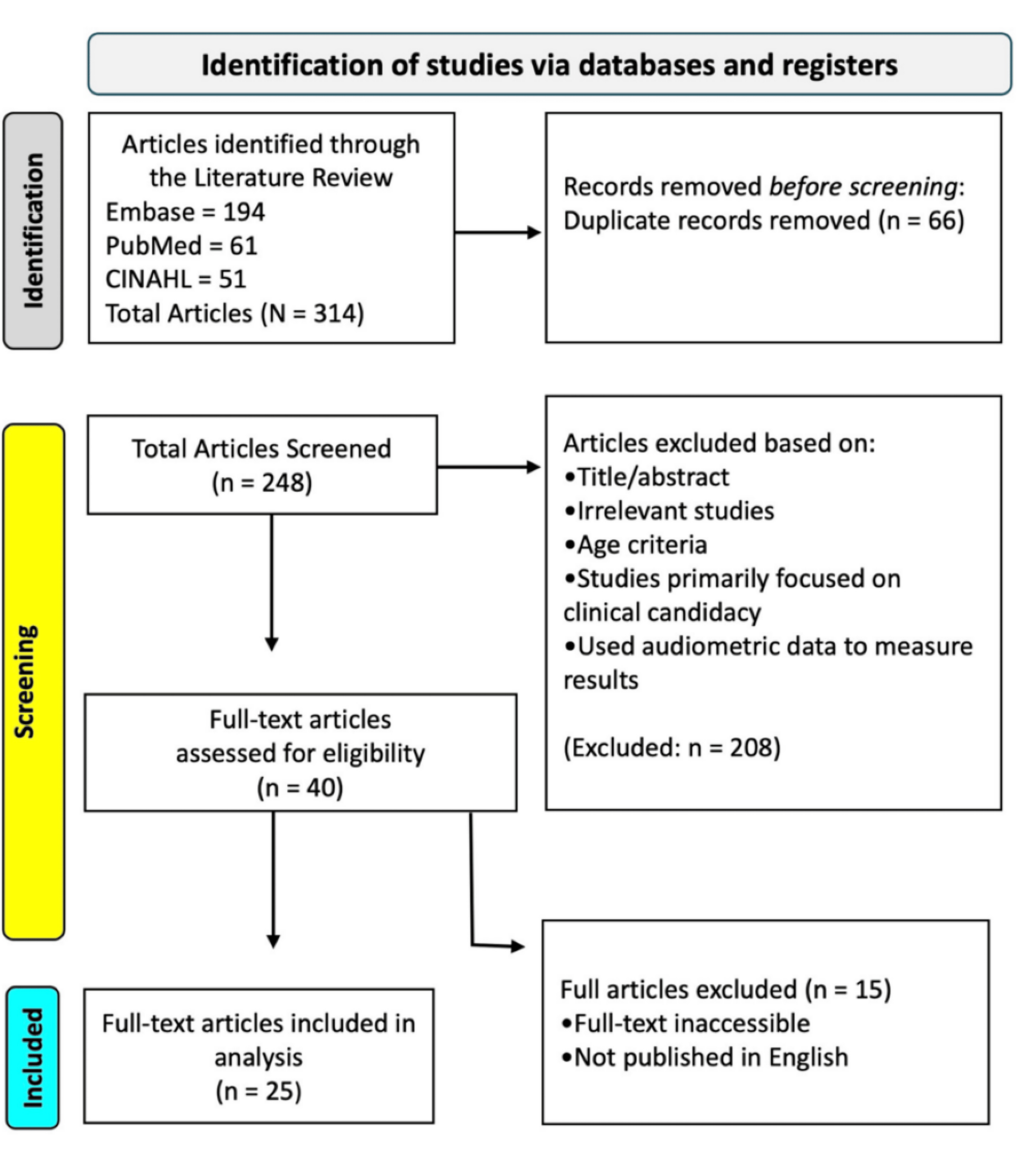
The Preferred Reporting Items for Systematic Reviews and Meta-Analyses (PRISMA) flowchart represents the study selection process.

This review was designed to explore the experiences and decision-making processes of parents and caregivers considering CI for their children. A narrative synthesis approach was used to synthesize qualitative and quantitative evidence on the psychosocial, emotional, and systemic influences on CI decision-making. The methodology was informed by established review standards and aligned with selected elements of the Preferred Reporting Items for Systematic Reviews and Meta-Analyses (PRISMA) guidelines (Figure 1). Selected PRISMA elements, including transparent reporting of the search strategy, eligibility criteria, and study selection process, were used, while formal risk-of-bias assessment tools and quantitative synthesis were not applied due to the exploratory and thematic nature of the review.

Eligibility Criteria

Studies were included if they examined the experiences, attitudes, or decision-making processes of parents or caregivers of children aged 0 to 17 with sensorineural hearing loss who were eligible for or undergoing CI, including both prelingually deaf children and those implanted at later ages. Eligible populations spanned all cultural, ethnic, linguistic, and socioeconomic backgrounds. The concept of interest focused on parental decision-making, including emotional, psychological, cultural, and social influences; expectations and values; communication with healthcare professionals and educators; accessibility of CI-related educational resources; and post-decision satisfaction or regret.

Studies were considered from any healthcare setting globally, including tertiary care centers, private clinics, and community hospitals, across both high-resource and low-resource environments. Accepted study designs included qualitative approaches (e.g., interviews, focus groups, ethnographies), quantitative designs (e.g., cross-sectional surveys, cohort studies), and mixed-methods methodologies. Only full-text, peer-reviewed journal articles published in English between 2000 and 2025 were included. 

Exclusion criteria were applied to studies that focused solely on adult CI recipients, that reported technical or surgical outcomes without caregiver input, or that centered on populations in which caregivers were not the primary focus (e.g., studies targeting only healthcare professionals or educators). Studies involving children with severe developmental or intellectual disabilities were also excluded unless parental decision-making remained a major analytical theme. Editorials, commentaries, protocols without results, single case reports, and non-peer-reviewed or non-English publications were not eligible for inclusion.

Search Strategy

To identify relevant literature on factors influencing parental decision-making in pediatric CI, we conducted a structured search of three databases: PubMed, Web of Science, and CINAHL Complete. Searches were completed in May 2025 using a combination of controlled vocabulary and key terms focused on sensorineural hearing loss and caregiver decision-making. The following search strategy was applied in PubMed and adapted appropriately to other databases: (“cochlear implant” OR “cochlear implants” OR “cochlear implantation” [Title]) AND (“decision making” OR “decision-making” [All Fields]) AND (“parents” OR “caregivers” OR “mother” OR “father” OR “parent” [All Fields])

After removing duplicates, titles, abstracts, and full texts were screened using the inclusion and exclusion criteria described above. Screening decisions were documented in a tracking matrix, and articles that met the eligibility criteria were retained for data extraction and thematic analysis.

Quality and Bias Assessment

Although formal risk of bias tools were not used to exclude studies, methodological quality and transparency were considered during the synthesis process. Each study was appraised informally for relevance, clarity of research aims, appropriateness of design, sampling strategies, and depth of interpretation. The review prioritized studies with clear descriptions of participant populations and robust methodological reporting. Limitations such as small sample size, recall bias, or lack of transferability were noted in individual study summaries when applicable.

Data Synthesis and Analysis

A narrative, thematic synthesis was used to analyze the included studies and identify common patterns across diverse contexts and methodologies. Key information from each study - including study aims, methodology, participant population, and major findings- was extracted and summarized in a structured table to facilitate comparison. Themes were developed through close reading of the results and discussion sections, with particular attention to recurring concepts concerning emotional and psychological responses, cultural and familial values, systemic barriers, and communication with healthcare professionals. These themes were refined iteratively as new insights emerged, allowing for the integration of both shared and divergent perspectives across studies. The synthesis aimed to highlight areas of consensus, variation by setting, and notable gaps in the literature. Given the heterogeneity of study designs, populations, and outcome measures, meta-analysis or meta-regression was not undertaken, and a narrative thematic synthesis was deemed most appropriate.

Characteristics of the Selected Studies

The final review included 25 peer-reviewed studies published between 2000 and 2025. These studies were conducted across a range of countries, including the United States, Canada, Belgium, Japan, India, South Africa, Australia, Brazil, and the United Kingdom. Study designs were diverse, with most employing qualitative interviews or focus groups; others used cross-sectional surveys or mixed-methods approaches. Sample sizes ranged from small qualitative studies with 6-15 participants to larger surveys with more than 100 respondents. Thematic focus areas varied by study but commonly included parental emotions, cultural identity, healthcare communication, educational access, and perceived barriers to implantation.

Results

The following section presents a thematic synthesis of the literature on parental and caregiver decision-making in pediatric CI. Across the included studies, several recurring domains emerged: (1) emotional and psychological responses to the decision, (2) communication and guidance from healthcare professionals, (3) accessibility of information and educational materials, and (4) socioeconomic and structural barriers.

Emotional and Psychological Factors

Parents consistently reported a range of emotional responses to the decision-making process, including anxiety, fear, guilt, and uncertainty. Across the studies that measured emotional burden, at least eight of the included articles explicitly documented high levels of parental stress, and in several of these, most participating parents described feeling anxious or overwhelmed by the permanence of the decision [5-7]. In studies that examined grief or adjustment to the diagnosis, caregivers in three to four studies reported unresolved grief as a factor that slowed or complicated their readiness to pursue implantation, indicating that emotional processing often preceded decision-making. Harding and Wright likewise reported that parents often balanced optimism about potential auditory gains with ongoing worries about surgical risk and the long-term impact on family life, reinforcing the emotional complexity of these decisions [8].

Concerns about the child’s future identity, communication method, and social integration were identified across 10 of the 25 included studies. In qualitative studies with detailed parent interviews, a majority of parents in those samples raised identity-related questions, including how their child would navigate Deaf and hearing environments and whether implantation might influence their sense of belonging. These themes were especially prominent in studies focused on bilingual/bicultural contexts or parental reflections on communication choices [9-11].

Communication and Support From Healthcare Providers

The clarity, consistency, and empathy of healthcare communication were cited as major influences on parental decision-making. Studies noted that parents often encountered differences in how providers explained candidacy criteria, expected outcomes, and rehabilitation demands, a theme reported in at least 6 included studies [5,12,13]. In several studies, audiologists, surgeons, and speech-language pathologists emphasized different aspects of the implantation process, leading some parents to perceive the information as fragmented. For example, Chang reported that parents received conflicting explanations of what constituted *successful* outcomes, while Archbold et al. found that families received variable guidance regarding expectations for post-implant therapy [5,13].

Additional inconsistencies arose regarding projections of long-term outcomes, with some clinicians offering optimistic expectations and others expressing greater caution. Several studies have also described parents receiving variable guidance regarding communication pathways, such as the anticipated roles of spoken language, sign language, or combined approaches, which left families unsure about how implantation might influence their child’s eventual communication identity. Together, these differences in emphasis and messaging reinforced parents’ perceptions that the information they received was incomplete or contradictory, thereby amplifying emotional and decisional stress.

Others emphasized the need for culturally sensitive counseling, particularly around Deaf identity and non-oral communication pathways, as a source of support rather than stress [9,14]. In these studies, culturally sensitive counseling involved acknowledging Deaf culture as a valid linguistic community, framing implantation without pathologizing deafness, and presenting sign language as a legitimate option alongside oral communication. Across all studies that examined this theme, each reported increased parental trust and reduced decisional conflict when clinicians offered clear and aligned explanations about candidacy, surgical risks, and long-term rehabilitation needs [6,15,16].

Accessibility and Readability of Educational Materials

Three studies examined how the design and delivery of written or digital materials influenced parental understanding and confidence. La Scala et al. found that most online brochures exceeded recommended reading levels, creating confusion and hindering decision-making, particularly for caregivers with lower health literacy [17]. Similarly, Hyde et al. and Dillon and Pryce noted that educational materials were rarely tailored to the needs of families from different linguistic and communication backgrounds, including households where caregivers were hearing, Deaf, or part of mixed Deaf-hearing families [18,19]. Some studies also reported on families who used more than one language at home, although bilingualism itself was not a primary focus of most analyses. When educational resources were accessible, comprehensive, and framed in plain language, parents expressed more confidence in navigating the CI process, defined in these studies as the full continuum of decision-making surrounding pediatric sensorineural deafness-from initial diagnosis and candidacy evaluation to discussions of communication goals, surgery, and the long-term rehabilitation pathway [5,17-19]

Socioeconomic and Structural Barriers

Across regions, financial strain, insurance limitations, and logistical barriers were consistently reported as challenges to timely implantation, appearing in at least seven of the included studies. In South Africa, families described long travel distances and inconsistent government funding as major obstacles [14], while parents in Brazil similarly reported that limited access to specialized follow-up care made the process financially difficult [7].

Hidden or ongoing costs also contributed to delays across both high-resource and low-resource settings. Families frequently cited expenses such as transportation to implant centers, ongoing therapy, and routine device maintenance as significant burdens, particularly for low-income or rural households [20,21]. Several studies noted that the price of batteries, processor repairs, or replacement parts could accumulate quickly, adding strain even when the initial surgery was publicly funded [20]. These financial pressures were further intensified for families facing language barriers or limited familiarity with healthcare systems, who often required additional time and support to navigate coverage processes [6,11].

Influence of Cultural and Community Perspectives

A subset of studies explored how cultural norms, religious beliefs, and extended family opinions shaped parental decisions. In several contexts, families reported seeking advice from Deaf community leaders, elders, or spiritual advisors, which influenced their comfort with or resistance to implantation [9,11,13]. In a subset of cases, multidisciplinary teams and families ultimately decided against implantation when anticipated benefit, family readiness, or long-term support needs were uncertain, highlighting that even positive candidacy evaluations do not always lead to surgery [22].

In some cases, the desire to *normalize* the child within mainstream society motivated early implantation, while in others, exposure to Deaf role models led families to explore alternatives such as prioritizing sign language, enrolling in Deaf-education programs, or delaying surgery to allow the child to develop within the Deaf community [5,10,23]. These sociocultural influences often intersected with healthcare communication, creating moments of internal conflict for parents seeking to reconcile conflicting messages. Archbold and Mayer further emphasized that educational context itself often serves as a major driver of parental decision-making, noting that families frequently linked implantation to broader hopes about their child’s long-term academic access, communication identity, and social integration [24].

Discussion

The findings from this narrative review reinforce the growing understanding that parental decision-making around pediatric CI is shaped by a range of emotional, social, informational, and structural factors. While early literature on CIs often prioritized surgical candidacy and audiological outcomes, more recent studies emphasize the complex, often nonlinear pathways that families navigate when making this high-stakes decision. These findings align with broader principles of shared decision-making and family-centered care, in which parental values, health literacy, and trust in healthcare providers play central roles in complex medical decisions.

Early work by Kluwin and Stewart described parents seeking repeated reassurance and additional information before agreeing to implantation, a pattern that continues to appear in more recent studies despite advances in technology and counseling [25]. Several studies emphasize the complexity and emotional weight involved in early decision-making for CI. Hyde et al. and Johnston et al. both describe the uncertainty families experience as they balance optimism for their child’s auditory development with concerns surrounding identity, surgical risks, and long-term outcomes [6,18]. Many parents reported feeling unprepared for the level of rehabilitation and ongoing responsibilities required after surgery, pointing to a gap between clinical counseling and the lived realities of families navigating this process.

Socioeconomic and healthcare system factors also remain influential. Ibrahim et al. found that ongoing financial strain, including costs related to travel, follow-up care, and rehabilitation, directly affected families’ decisions to pursue implantation [21]. Similarly, Peñaranda et al. described the long-term emotional and logistical challenges families encountered when coordinating follow-up care and managing the practical demands of the implantation process [26]. Together, these studies highlight the need for a more equitable and sustained model of care that extends beyond the surgical procedure itself.

Family structure and expectations further shape the decision-making process. Hyde et al. described how parental stress, competing family responsibilities, and differing caregiver perspectives contributed to delays or uncertainty during decision-making [27]. Chang emphasized that family dynamics influence both the timing of implantation and the degree of engagement in postoperative care [5]. Families with other deaf children or deaf relatives often brought prior knowledge, communication strategies, and cultural perspectives that informed more deliberate decisions. In contrast, parents of an only deaf child with no prior exposure to deafness tended to rely heavily on clinical guidance and reported greater uncertainty following implantation.

Cultural identity and community alignment continue to play a defining role in how parents frame their choices. McMenamin et al. examined how family attitudes, available resources, and cultural orientation influenced children’s acculturation experiences following CI [28]. Some families prioritized spoken language integration, while others sought a balanced approach that preserved visual language exposure. Similarly, Peñaranda et al. used narrative interviews to explore how parents made sense of the implantation process, with many describing ambivalence when their child’s outcomes did not align with initial expectations [26].

Device-related factors also emerged as meaningful contributors to decision-making. Clamp et al. found that aspects such as device design, size, aesthetics, and perceived reliability shaped parental and child preferences, at times leading to differences between clinical recommendations and family priorities [29]. Dillon and Pryce echoed this, noting the growing influence of peer families and online communities in shaping parental expectations and final decisions [19].

Finally, studies such as Archbold et al. and Peñaranda et al. underscored the importance of comprehensive support that extends well beyond surgery [13,26]. Parents consistently expressed a need for emotional guidance, opportunities to connect with other families, and practical strategies for supporting early development following implantation.

Taken together, these studies broaden the understanding of CI as not only a medical intervention but also a deeply personal and culturally situated decision. They reinforce the importance of tailoring support to each family’s unique context, values, and evolving needs, and of ensuring that future research and clinical practice reflect this multifaceted experience.

## Conclusions

This review demonstrates that parental decision-making for pediatric CI is influenced by a combination of emotional, informational, cultural, and structural factors. While the clinical benefits of early implantation are well established, parents often face uncertainty, inconsistent guidance, and practical barriers that shape how they evaluate the procedure and its long-term implications. The evidence highlights the importance of clear communication, culturally responsive counseling, accessible educational resources, and reliable follow-up services in supporting families during the decision-making process.

Future research should continue to examine how these influences vary across cultural, linguistic, and socioeconomic contexts and evaluate strategies to improve the clarity, consistency, and accessibility of information provided to families. Efforts to assess models of care that reduce logistical and financial burden, such as telehealth-based counseling or integrated care pathways, may help address persistent structural barriers. Collectively, these findings underscore the need for family-centered approaches that acknowledge the complexity of decision-making beyond clinical candidacy and support equitable access to CI services.
